# Resident-, family-, and staff-identified goals for rehabilitation of long-term care residents with dementia: a qualitative study

**DOI:** 10.1186/s12877-024-04674-2

**Published:** 2024-01-29

**Authors:** Sara Ripley, Niousha Alizadehsaravi, Rebecca Affoo, Susan Hunter, Laura E. Middleton, Elaine Moody, Lori E. Weeks, Caitlin McArthur

**Affiliations:** 1 Dalhousie University, 5869 University Avenue, B3H 1X7 Halifax Nova Scotia, Canada; 2 Western University, 1151 Richmond Street, N6A 3K7 London, ON Canada; 3 University of Waterloo, 200 University Avenue West, N2L 3G1 Waterloo Ontario, Canada; 4 Schlegel-UW Research Institute for Aging, 250 Laurelwood Drive, N2J 0E2 Waterloo, ON Canada; 5 Centre of Excellence, Aligning Health Needs with Evidence for Transformative Change (AH-NET-C): A JBI, Dalhousie University, 5869 University Avenue, B3H 1X7 Halifax Nova Scotia, Canada

**Keywords:** Rehabilitation, Dementia, Long-term care, Physical therapy, Occupational therapy, Goals, Function, Quality of life

## Abstract

**Background:**

Long-term care (LTC) residents with dementia can benefit from rehabilitation to improve function and quality of life. However, specific goals for rehabilitation with this population are not always clear. The purpose of this study was to describe the goals for rehabilitation for LTC residents with dementia from the perspective of residents, family, and staff.

**Methods:**

This was a phenomenological qualitative study. LTC residents with moderate to severe dementia, family members, and staff were recruited from two LTC homes in Halifax, Nova Scotia. Data were collected through semi-structured interviews and field notes from observations with residents while they were being active within the home. Data were analyzed via the principles of thematic content analysis, mapped onto the International Classification of Functioning, Disability, and Health (ICF) Model, and reported by the participant group (i.e., residents, family, or staff).

**Results:**

The 15 participants were three female residents aged 82 to 98 years, seven predominantly (86%) female family members aged 56 to 74 years, and five staff members (two females, three males, aged 22 to 55 years) who were physiotherapists, a physiotherapy assistant, a healthcare aide, and a registered licenced practical nurse. Most identified goals fell within the activities and participation constructs of the ICF model and focused on maintaining or improving function, mobility, and quality of life. Specific themes included preventing falls, walking or locomoting, stair climbing, maintaining activities of daily living, engaging in enjoyable exercise, maintaining independence and human connections, keeping busy, leaving the home for activities, and participating in group activities.

**Conclusions:**

Rehabilitation goals for LTC residents living with dementia often focus on quality of life and functional activities and participation in LTC and family activities and events. Function and quality of life are interrelated, whereby functional goals influence quality of life. While some goals focus on improvement in function, maintenance or prevention of decline were also key elements. Future work should ensure rehabilitation interventions are developed relative to individually identified goals, and interventional success is measured in relation to the goal.

**Supplementary Information:**

The online version contains supplementary material available at 10.1186/s12877-024-04674-2.

## Background

Dementia is a neurological condition whose prevalence increases with age and which results in physical and cognitive decline [1]. Dementia is prevalent in long-term care (LTC) homes, facilities that provide health and personal care for people living with medical or physical needs who require access to 24-hour nursing care, personal care, or other therapeutic and support services [2]. As many as 80% of LTC residents have a diagnosis of dementia [3], and the number of newly admitted residents with dementia is increasing [4]. LTC residents with dementia experience high levels of disability, including functional dependence and an increased risk for falls and fractures and reduced quality of life [3, 5–8].

Rehabilitation can help maintain and improve function for LTC residents living with dementia [9]. However, LTC residents with dementia are less likely to receive rehabilitation services than those without [10]. Rehabilitation providers often become frustrated when they do not achieve the anticipated results when working with individuals with dementia [11]. Yet, many rehabilitation interventions (e.g., group exercise classes) are not customized to draw on the strengths and unique needs of individuals with dementia. Further, there is limited evidence to support physical interventions for people with dementia living in LTC [12] and with more advanced dementia [13].

Previous work suggests that setting goals is an essential component of rehabilitation [14–16] and with people living with dementia [17–19]. Goal setting may result in improved psychosocial outcomes (i.e., health-related quality of life, emotional status, and self-efficacy), though it may not improve physical outcomes [20]. Despite its importance, Hall et al. found a lack of goal setting for rehabilitation with people living with dementia which led to challenges, confusion, and dissatisfaction by those living with dementia and their informal caregivers [21]. Goal setting relative to dementia has been suggested to be less clear and not well-defined [17] which could be because it is often perceived as an incurable disease [21]. However, goals should be carefully delineated relative to the condition for which the person living with dementia is receiving treatment rather than in pursuit of curing the overall disease [13, 21].

Levack et al. identified goals for rehabilitation for a wide variety of conditions such as stroke, musculoskeletal and chronic pain conditions, mental health, age-related disability, and cardiovascular or respiratory conditions [20]. Goals included those related to body structure and function, activity limitations, participation restrictions, functional goals, dietary behaviour, amount and frequency of practice of coping skills, and emotional, financial, and information needs [20]. Two of these studies were conducted in LTC homes [22, 23] and focused on goal setting to decrease functional dependency for residents. Importantly, none of these studies focused specifically on the goals of rehabilitation for LTC residents with dementia which may be different than other settings or health conditions. Given the progressive nature of dementia and the fact that LTC residents are often within the last few years of their lives [24], goals may focus more on maintenance of function and quality of life rather than improvement or discharge home. However, goals of rehabilitation for LTC residents with dementia as identified by residents, family members, and staff have not been examined in the available literature to date.

Therefore, the purpose of this study was to describe the goals of rehabilitation for LTC residents with dementia from the perspective of residents, family members, and staff. With goals identified, interventions can be designed to achieve those goals and subsequently tested. Further, the results can support advocacy for access to rehabilitation in pursuit of the identified goals. Indeed, previous work suggests that the culture of the organization must support person-centred rehabilitation for access to be optimized for those with more advanced dementia [21], like in LTC.

## Methods

A phenomenological qualitative methodology was used to explore goals related to rehabilitation for LTC residents with dementia. This study is written in accordance with the consolidated criteria for the reporting of qualitative research (COREQ) (See Supplemental File 1 for checklist) [25].

### Ethical approval and consent to participate

This study was reviewed and approved by the Dalhousie University Social Sciences and Humanities Research Ethics Board. All methods were carried out in accordance with the relevant guidelines and regulations. Informed consent was obtained from all participants or their legal guardian. Assent was also obtained from those who could not provide consent for themselves.

### Participants

Three groups of participants were included as follows: LTC residents with moderate to severe dementia, family members, and staff members. Participants were purposively recruited from two not-for-profit LTC homes in Halifax, Nova Scotia. Both homes were in an urban area with 385 and 156 beds, respectively. LTC residents with moderate to severe dementia with a Mini-Mental State Exam ≤ 20 were included in the study to accurately reflect the level of cognitive impairment with which most LTC residents live. Residents receiving palliative care or active end-of-life care were excluded from the study as their goals for rehabilitation may be different. Eligible family members had a relative in one of the two LTC homes from which participants were recruited, while eligible staff had at least 6 months of LTC experience and provided direct care to residents with dementia at the LTC homes of interest. All participants were able to be interviewed in English. Recruitment occurred over a 4-month period between December 2022 and March 2023, until data saturation was reached. A research assistant at the LTC homes sent out an email with the study flyer and information to resident’s substitute decision makers (e,g,, family members) and staff members. Passive recruitment also occurred whereby the flyer was placed on television monitors in common spaces through the LTC homes. The research assistant at the LTC home also emailed floor managers directly to help recruit staff members. Those substitute decision makers and staff members who expressed interest emailed or called the principal investigator. Following the determination of eligibility, researchers reviewed the consent forms with the participants and obtained informed consent. Before data collection, participants had either met or engaged in discussions with the research team, cultivating a connection that facilitated collaboration throughout the study.

### Data collection

Semi-structured interviews and observations were conducted with LTC residents with moderate to severe dementia, their family members, and staff working closely with them. To enhance the interview experience with LTC residents with communication challenges, all interviews with residents were carried out using a dyad format (*n* = 3), which included both residents and their family members. In the dyad format, the resident and their family member participated in the interview at the same time. Questions were asked to both the resident and their family member following the appropriate semi-structured interview guide. During the dyad interview sessions, residents were asked the questions from the resident version of the interview guide and answered with support from their family members. If a family member stated a goal for the resident, the interviewer would confirm with the resident that this was indeed their goal. Within the same session, family members were also asked the questions from the family member version of the interview guide to ensure we captured both points of view. Some family members (*n* = 4) participated in individual interviews without a resident because their loved one was unable to participate in an interview. To minimize risks or discomforts for LTC residents, interviews were conducted within a comfortable space within the home based on their preferences (e.g., their room, common room). Ongoing assent was established throughout the interview and if the resident expressed any wishes to discontinue the interview was stopped. Staff interviews were conducted individually. Interviews were conducted by the principal investigator, a trained physiotherapist and researcher who has eight years of experience conducting interviews for qualitative research, and who identifies as a woman. Participants had information about the interviewer’s gender, credentials, and experience prior to the interviews. Interviews lasted between 30 and 60 min and followed semi-structured interview guides designed specifically for each of the three groups of participants (Supplementary File 2). The semi-structured interview guides were developed in consultation with a project advisory committee consisting of researchers, clinicians, and patient and public partners with lived experience with dementia and the LTC setting. All participants were asked to provide their perceptions of the goals of rehabilitation in the LTC homes. For residents who couldn’t communicate, family members acted as a proxy (*n* = 1), providing responses on behalf of their loved ones, and with respect to the population in general, while staff were asked to answer relative to the population. The interviews were audio-recorded and transcribed using otter.ai software (Otter.ai, Inc., Mountain View CA). A research assistant established accuracy of the transcripts by comparing them with the audio recording. For LTC residents, an observation was conducted by the principal investigator where field notes were taken during a time when the resident was active or doing activities they enjoyed (e.g., dancing, walking, participating in seated exercises, playing Bingo). The observations were guided by a semi-structured field note guide (Supplementary File 2) and scheduled with the family member who participated in the interview present. The observations provided an opportunity to understand the context of the experiences of LTC residents in relation to the research questions and for participants who were not able to communicate verbally to participate.

### Data analysis

Data from both interview transcripts and field notes from observations were analyzed by research assistants using the principles of inductive qualitative description and thematic content analysis using Clarke and Braun six step guide [26] to identify themes describing the goals of rehabilitation and the meaning of quality of life and function. Systematic coding was carried out by inductively coding the transcripts in NVivo 1.6.1 (Lumivero, Denver CO). As themes and subthemes surfaced from the transcripts, they were integrated into the developing codebook. Two research assistants coded the data independently. The research assistants met frequently with the principal investigator to discuss the results, and the research team reviewed and provided input on the final themes to ensure validity of the results. The authors of this article are a multidisciplinary group of researchers and clinicians focused on geriatrics, dementia, exercise, rehabilitation, and LTC. Therefore, the authors could have preconceived ideas of the rehabilitation goals for LTC residents and highly value rehabilitation provision for this population. However, thematic analysis was conducted independently by two research assistants who are not rehabilitation providers in LTC limiting the potential for biases to influence the results.

**Fig. 1:**
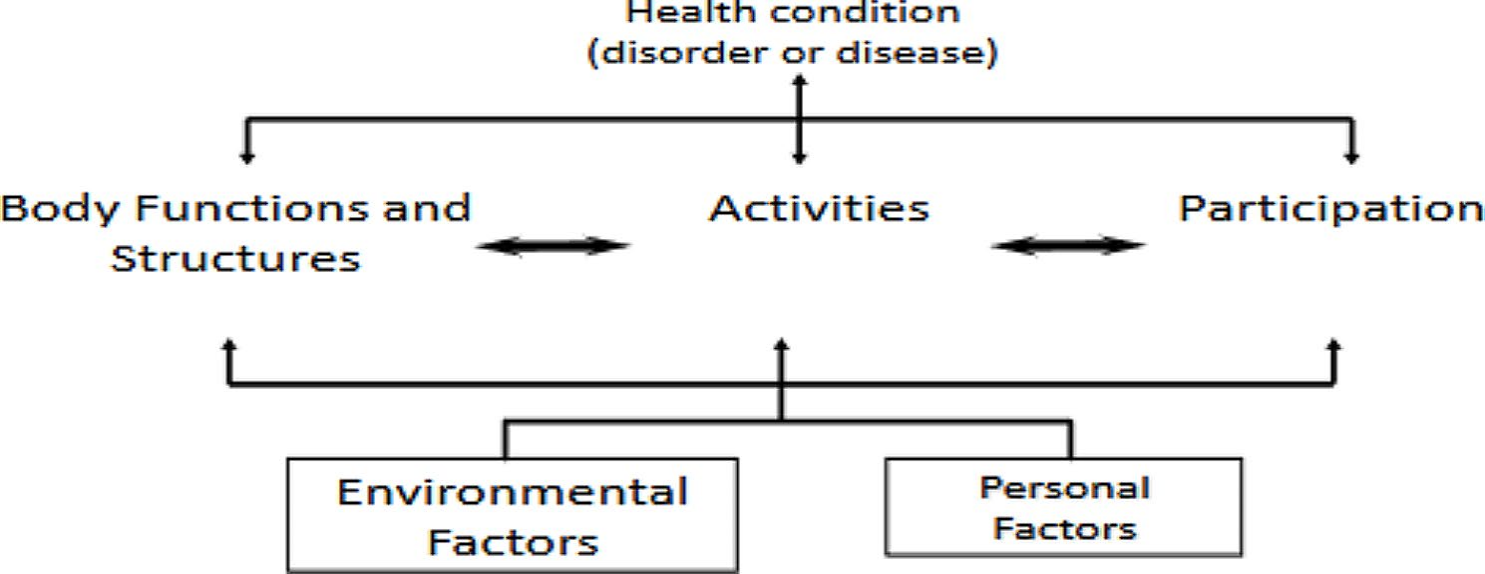
International classification of functioning, disability, and health [27]

To represent all participants’ voices, results are presented by participant group (i.e., resident, family member, and staff). The International Classification of Functioning, Disability, and Health (ICF) Model was used to conceptually organize the themes arising from the results (Fig. 1). Use of ICF model, as elucidated by Levack et al., serves as a robust foundation for organizing the emergent themes by providing a standard language and conceptual basis for the definition and measurement of health and disability and provides a framework for organizing and describing information on function and disability [27]. The ICF posits that disability is multidimensional and interactive where functioning at the level of the body (i.e., physiological function and anatomical parts of the body), activity (i.e., execution of a task or action by an individual), participation (i.e., involvement in a life situation), and the environmental (i.e., physical, social, and attitudinal) and personal factors which influence these experiences all interplay to affect a person’s level of functioning [27]. After finalizing the themes, the researchers mapped them onto the ICF model. In the context of identifying rehabilitation-related goals, the themes were observed to align primarily with the body function and structures, activity, and participation domains of the ICF model. Following analysis, there was no explicit discussion of environmental and personal factors within this context. Once the themes were identified, a summary of findings was shared with the participants to obtain their feedback and ensure the results accurately represented their point of view.

## Results

Out of the five residents who expressed interest in the study, one was determined to be ineligible for participation due to receiving active end-of-life care, and the other resident had mild dementia (MMSE = 28). The remaining three residents were female and between the ages of 82 to 98 years. Residents lived in LTC for 2 to 4 years and had dementia for approximately 7 to 8 years. There were seven family members of residents with dementia in LTC involved in the study, including four children, two nieces, and one spouse/partner of residents. Eight family members initially contacted the researchers to participate, but following eligibility identification by the researchers, one withdrew their interest in participating. Most of the family members were female (*n* = 6), with their ages ranging from 56 to 74 years. The residents related to the family members had dementia for 5 to 21 years and lived in LTC between 2 and 15 years. Five staff members working in LTC participated in the study, two females and three males between 22 and 55 years of age. Two of the staff members were physiotherapists, one was a physiotherapy assistant, one was a healthcare aide, and one was a registered licenced practical nurse. The staff members reported working in LTC for 3 to 20 years.

**Fig. 2:**
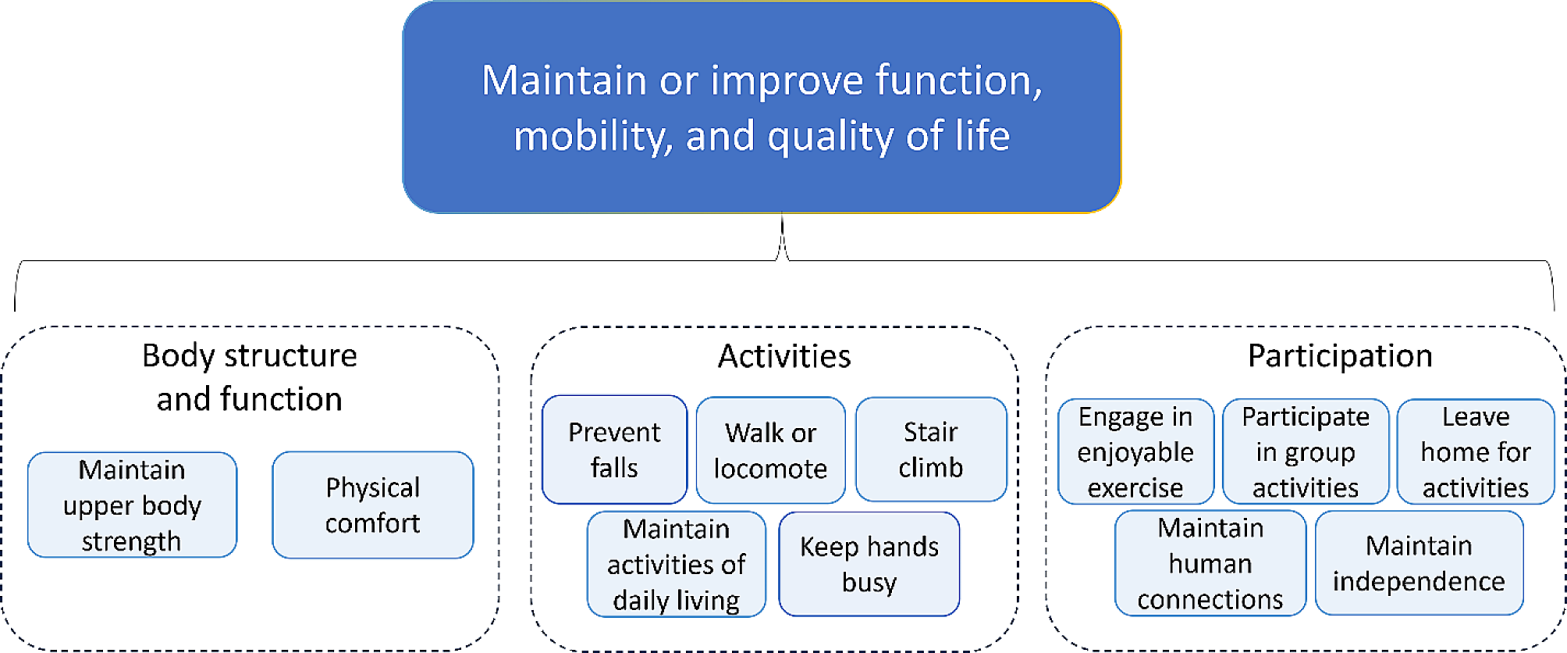
Rehabilitation goals for residents with dementia in LTC

### Overarching theme: maintain or improve function, mobility, and quality of life

Figure 2 provides an overview of the results derived from the analyses conducted in this study. Maintaining or improving function or mobility was a central goal for rehabilitation for residents with dementia in LTC. Family and staff members expressed the importance of staying active, indicating that it allows residents to do things they enjoy such as leaving the home for an activity with family, or going for a walk either outdoors or around the facility. Residents described wanting to be able to do activities like sit up, stand, and walk so that they could participate in activities within and outside of the home. Further, family members mentioned that maintaining circulation and blood flow while being mobile and active is very important especially in residents with comorbidities such as heart conditions.

Although maintenance of function was at the forefront of many of the participant conversations, staff members also mentioned regaining lost function as a goal for rehabilitation for residents. This could be function that was lost because of dementia, or because of injury or comorbidity. Residents mentioned regaining lost function such as the ability to sit independently or walk. When discussing progressive walking and strength programs, one staff member describes:

Within that it is different goals, and the goals then are dependent on the acuity of what the resident is experiencing. If someone has had a tumble, they’re a little bit sore, or if they’ve had a urinary tract infection and they’ve been off their feet for a little while, can we help them regain what they recently lost? And giving them that support to do that on a regular basis can be sometimes successful.

Improvement of quality of life is a goal for rehabilitation that was discussed by both staff members and family members. Participants remarked that this does not necessarily mean slowing the progression of the dementia, but instead making the resident feel as comfortable as possible throughout their stay in LTC. One staff member described this idea:

…rehabilitation won’t necessarily mean a decrease in the progression, but it could just mean living a better life, having a better quality of life in the different stages that they’re at. I mean, if you can avoid a wheelchair that, for some people, can often give them better quality of life, no matter what stage they are in dementia. That’s, I think, a big one. And I think part of the rehabilitation is improving the quality of life. I mean, just about all of them want to be home and don’t really want to be here. But if you can make it as pleasant as possible, then that’s OK.

The findings revealed an interrelationship between function and mobility and quality of life. Function and mobility were deemed important for good quality of life by family members of residents and staff, as well. One family member speaks to the benefit of having even minimal mobility. For example, if the resident is mobile enough to be able to transfer to a wheelchair, there are then more opportunities for family members to take residents out of the LTC home for activities and gatherings. Further, this staff member speaks to the role of mobility in being comfortable from a physiotherapist’s perspective:

*“I just have this physio lens hat on right now, so thinking of movement, but if you’re just sitting there all the time, it’s painful, so you experience discomfort. So there’s this relief coming from movement that you have just being able to move, lubricating joints and have a change of position. That’s an aspect of quality of life, just being comfortable.”*

### Subtheme: body structure and function

#### Maintain upper body strength

Maintaining upper body strength was mentioned by family members as a goal. This was especially important in the context of independent wheelchair propulsion and in encouraging residents to participate in rehabilitation with family members. For example, one family member described:

We started doing exercises with her so she could get her arm above her head and throw. She was a baseball player, so we could get her to throw soft, squishy balls at us and get her arms and her hands moving because we knew it was important for her to have some mobility in her hands and arms.

### Physical comfort

Family and staff members both mentioned reducing pain as a goal. They mention reducing physical pain for the resident as much as possible, recognizing that the resident may still be in some pain despite rehabilitation efforts. When discussing fall and injury prevention as a rehabilitation goal for their mother, one family member emphasized the importance of pain reduction:

“.*and keeping her pain free. Keeping her pain under control as much as we can.”*

Several staff and family members noted that being comfortable also plays a significant role in the resident’s quality of life. The participants linked being comfortable with being pain-free, as well as being generally content. This staff member elaborates:

“*Are they comfortable? Pain-free.…I think those things are important.”*

### Subtheme: activities

#### Prevent falls

Fall prevention was mostly discussed by staff and family members. Discussion was primarily centred around the importance of maintaining strength and mobility to prevent falls, and how maintaining strength and mobility will also impact the effect a fall will have on a resident. Conversely, it was noted that with fall prevention as a main goal for rehabilitation, physical function is often sacrificed in the interest of resident safety highlighting a challenging balance between autonomy and safety. As one staff member notes:

With fall prevention quite commonly as a goal, how do you balance fall prevention with quality of life? That to me is also a really challenging ethical question. When you’re balancing that living at risk. When you’re trying to help somebody physically function better, but they don’t necessarily have the insight that their issues of cognition and impulsivity and overestimating their own abilities puts them at constant risk. Where do you draw the lines on these types of things for them? And it’s a moving target and an ongoing negotiation and level of comfort for those types of things.

### Walk or locomote

The ability to walk is a goal that was expressed by residents, family members, and staff members alike, and it was the most referenced activity within this theme. Walking was important for several reasons, including enjoyment, quality of life, and practicality. For example, a family member described that it was important for their spouse to be able to walk for enjoyment, as they had been an active walker for most of their adult life. Another family member described the benefits of being able to walk in a practical sense, as, if the resident can walk even a minimal distance it could facilitate the transportation of the resident outside of the LTC home into the family member’s home or move around the home, for example, to meals. Where walking was no longer possible, self propulsion in a wheelchair was also described. As one of the staff members described:

*“I find the biggest one is just being able to walk. For example, we have a physiotherapist and they take them to the gym and they get the legs still moving. I think that’s most goals, is just to keep them mobile as long as we can because I find what happens is they are such a high risk for falls that just, nice that we can keep them walking as long as we can or if they’re in a wheelchair moving their legs and that around.*

### Maintain activities of daily living

Several staff members emphasized the importance to residents of maintaining activities of daily living. As one staff member noted:

I think just maintain the abilities, physical abilities to continue doing activity of the daily life. Stand up, sit down, walking, maybe roll, roll transfer in bed. From my job position and perspective, I think that is the main focus of rehab.

The activity of daily living the staff members mentioned most was toileting. They also discussed the specific activities involved in toileting independently, such as donning and doffing of clothing and performing basic personal hygiene. This staff member speaks to how toileting fits into rehabilitation:

Toileting is a good example. There’s toileting programs where we can do things to prevent them from being incontinent. So, it would be like getting them set up so they’re being toileted every two to three hours…. Sometimes they come here completely incontinent from the hospital because, as we know, their focus is very different than ours. So, we get them here and then we start toileting them all the time. And some of them do end up not being incontinent anymore from it, for a while.

Other activities of daily living that were discussed were the ability to sit up, stand, and transfer to a bed. Sitting up is important for resident comfort and especially for eating and drinking. One resident described that they would simply like to be able to comfortably sit in their rocking chair and relax. Another resident, who expressed having more difficulty sitting up on their own, described being able to sit up as her primary goal for rehabilitation. When asked what her goal was, the resident stated, “*I can’t sit up”.* The interviewer asked if she would like to be able to sit up, to which she responded *“yes”*. This resident was observed having difficulty maintaining upright posture during activities throughout the home such as bingo and during meals.

Both staff and family members commented on how being able to stand and weight bear is important to residents. Staff members described that the ability to stand, with or without an aid, is necessary in maintaining many functional movements such as accessing the toilet. This family member describes helping their mother perform leg and hip strengthening physio exercises to maintain her ability to stand:

I was doing leg raises with Mom in her wheelchair to keep her quads strong because we knew they needed that for her hips to get her to stand. So, having that program early on is really important.

Being able to transfer to a bed is an important goal for residents, as described by staff members. If a resident possesses the ability to transfer to a bed, it allows them more freedom to leave their bed and to potentially leave their room on their own terms throughout the day.

Additionally, staff and family members mentioned the ability to do activities of daily living as an indicator for quality of life. This staff member relates being able to do activities of daily living to autonomy and highlights the importance of being able to toilet, specifically:

…being able to use the bathroom. I think for some residents, when they have to move to a sling lift or a bed pan or even just a pad that that can be really hard and not ideal for them, so being able to maintain that ability to use the bathroom is a quality of life thing.

### Stair climb

Stair climbing was raised as a potential goal for rehabilitation, particularly as it relates to having the ability to leave the LTC home to visit with family. If the resident’s goal is to be able to leave the LTC home for a family outing or event, then they need to be prepared to function in a potentially unfamiliar environment, as this staff member describes:

We do even get into sometimes ability to stair climb, and those kinds of accessibility issues. Things that are very challenging from long-term care because we don’t do that typical home visit, discharge planning, and often these sorts of visits that are coming up at Christmas times and big celebrations or funerals or things like that are going into unfamiliar environments for everybody.

### Keep hands busy

Family members and residents described keeping their hands busy as a goal for residents with dementia in LTC. A resident mentioned that it is especially important to them to have something to do with their hands because they mostly stay in their bed or chair. During the observation, residents were often completing tasks to keep their hands busy such as folding towels, completing puzzles, or creating arts and crafts. A family member outlines the importance of aiding residents in keeping their hands busy early, so that it will be easier to maintain as the disease progresses:

Unfortunately, I think what the key for a lot of patients with dementia is you gotta get them early to using their hands. So they have some connection to their body. And with dementia, they lose their use of words and language, but you can cue them like I could cue mom a lot with her hands in different things to get her to pick up a pencil.

### Subtheme: participation

#### Engage in enjoyable exercise

Participants emphasized the ability to exercise as a goal, including all forms of exercise within the resident’s capacity such as using a bike in the fitness centre or attending seated exercise classes. Residents were observed participating in group exercise that included enjoyable movements like seated dancing. One family member expressed the ability to dance as a goal for rehabilitation, as it is an activity their spouse has always enjoyed:

In the early part of her stay here, she’s always loved dancing….For the first few months, we would always go to that and this was lovely for her. But [dancing] was something of a form of physical activity that she’s always enjoyed. So, for her for a time that was also important.

### Maintain human connections

Family members expressed maintaining human connections as a goal for rehabilitation where the benefits of rehabilitation enable the residents to engage in social activities. One family member remarked that the rehabilitation process itself was a social activity for the resident:

So, that social aspect too, so just having human contact, be it with a physio, a nutritionist, a nurse, a personal support worker, is huge to me.

Further, maintaining connections was the most frequent answer to the question of what quality of life means to the resident. Most commonly, both family and staff members referred to connecting with family and friends, either with in-person visits or phone calls. However, some participants mentioned maintaining connections with other residents and even staff members, as well. This family member describes her family’s joint effort in ensuring their mother receives adequate socialization:

*“She loves to be with people. She loves visitors. She likes to get phone calls.… She likes to sit and chat with the other table mates and things. I like to see her more interacting with other people in the unit, not necessarily at a group thing. Just sitting the dining room, chit chatting.”*

### Maintain independence

Both family and staff members indicated that maintaining independence is important to residents. One staff member noted that it is a goal of residents to prevent decline in their autonomy and independence. From another staff member’s perspective, the idea of maintaining independence motivates residents to engage in rehabilitation, and the same staff member described its significance relative to quality of life:

…can we set somebody up in an equipment that they can get around by themselves? If there’s any way that we can maintain that little bit of independent mobility, I think that adds to a quality of life. If somebody can move their chair across the room to get the drink of water without having to call somebody, that’s very significant.

Participants noted that many residents can maintain at least some level of independence regardless of the stage of their dementia. For example, for some residents being independent could mean being able to walk, while for others it could mean being able to use the TV remote or being able to access the call button.

### Leave the LTC home for activities

Some of the staff members emphasized how important it is to some residents to be able to leave the LTC facility for activities. The staff members describe that significant barriers to achieving this goal are if the residents are unable to walk or if they are unable to get into and out of a vehicle. Another factor at play is that the staff members must effectively communicate the potential risk that traveling from the LTC facility may pose to the resident. This staff member describes the importance to residents of leaving the LTC home to visit family, especially around the holidays:

For some of them, a big one is still being able to go out with family and getting into and out of a car. Being able to do a couple steps into a house. I find especially around the holidays that’s a big goal for them to be able to do that.

Moreover, spending time outside of the LTC home was a common response to the question of what quality of life means to the resident, especially from family members. A couple of family members mentioned the importance of being able to leave the LTC home for hours to attend family gatherings, while others highlighted the benefit of getting fresh air in the garden at the LTC home, for example. As this family member states when asked about quality of life for her mother: *“Be able to get around a little bit or even just get outside once in a while for some fresh air.”* The resident participating in this dyad interview reported she enjoyed going out to the garden: *“And every day we would go up (to the garden) and take our coffee and a doughnut. And we’d sit by them and we’d almost see the flowers growing.”* Having the mobility to get to the garden was very important for this resident’s quality of life.

### Participate in group activities

Staff members describe that it is important to residents to be able to participate in group activities due to the mental and physical benefits. Not only are group activities often good exercise for the residents, but they also offer a social environment. Both family and staff members also emphasized the importance of participating in group activities for maintaining quality of life. Residents were observed participating in several group activities like music sessions and group exercise, which produced happiness and joy for the participants. One of the staff members referred to the social and physical benefits of group exercise programs, while this family member expressed the benefit of different types of group activities:

So, they do have activities here, which is great….She loves Bingo. And trivia….She used to do a lot of it when she first came in,

## Discussion

This qualitative study reveals that rehabilitation goals for LTC residents living with dementia often focuses on quality of life and functional activities and participation in LTC and family activities and events. There is an interrelationship between function and quality of life, whereby many functional goals also contribute to improved quality of life. While some goals may focus on improved function, maintenance or prevention of decline were also key elements of the identified goals. Rehabilitation interventions should be developed relative to an individual’s identified goals, and success of the interventions should be measured in relation to the goal.

Like the work by Levack et al. [20], many of the identified goals in this study were focused on the activities and participation domains of the ICF model. Specifically, functional activities like walking, standing, transferring, and toileting were common identified goals while participating in group activities and leaving the LTC home to visit family were common participation goals in this study. ADLs could be classified under both the Activities and Participation domains depending on the contextual factors of the task. Participants described ADLs as specific, tangible tasks (e.g., toileting, transferring) which aligns more closely with the Activities domain. Given the increasingly high levels of functional impairment for LTC residents [3, 4] and the progressive nature of dementia, results are not surprising. Despite their high levels of functional impairment [3], a relatively small amount of LTC residents with dementia receive rehabilitation [10]. The most identified goals in this study focused on functional maintenance, or improvement where possible, and improved quality of life. Establishing clear goals with people living with dementia and their caregivers can help prevent challenges related to rehabilitation, promoting clarity and satisfaction [17].

Participants in this study emphasized the importance of functional maintenance and prevention of decline in addition to improving function where possible. A 2013 systematic review and meta-analysis revealed a small effect of physical rehabilitation on activities of daily living for LTC residents, which the authors hypothesized may reflect maintenance of function and be appropriate for a population that tends to become increasingly frail and dependent [28]. Dementia is a progressive disease where function usually deteriorates over time, though the trajectory of deterioration varies [6]. Thus, prevention of decline is often a reasonable goal for this population. However, in some circumstances it may also be a reasonable goal to improve function, and this should not be disregarded as a potential rehabilitation goal. Excess disability, where functional decline is more than would be expected by the disease course, is common in dementia [11]. Careful consideration of where functional improvement may be possible is necessary and it should not be presumed that all LTC residents with dementia do not have the potential to improve. Further, falls prevention was an important goal identified in this study. Recent guidelines suggest that all LTC residents should be considered at high falls risk [29]. Thus, all clinicians should consider falls prevention when developing functional goals for individuals at high risk like those in LTC.

Results reveal an interrelation between quality of life and function, where positive changes in function were often linked with positive changes in quality of life. Indeed, many of the previously identified domains of quality of life in LTC overlap with the functional goals identified in this study. For example, physical comfort, functional competence, autonomy, meaningful activity, and relationships [30] were represented as goals of rehabilitation and reflecting good quality of life. Some challenges may exist in measuring self-reported quality of life for LTC residents with more advanced dementia as communication challenges could prevent an accurate representation. Those with mild to moderate cognitive impairment may be able to complete a self-reported tool [31]. However, for those with more advanced dementia, a proxy report may be required and has been found to be feasible [24].

Given the importance of quality of life in our study and its interrelationship with function, a common goal of rehabilitation, authors urge future researchers to measure quality of life as an outcome and clinicians to consider it as an outcome measure to guide their clinical practice.

This study adds to the current literature a comprehensive perspective of the goals of rehabilitation in LTC. Indeed, a strength of this study is the inclusion of the perspective of three important groups in the LTC sector: residents, family members, and staff giving a fulsome perspective on the topic. Of particular importance is the inclusion of LTC residents with dementia as they are often excluded from data collection. The research team adapted the data collection strategies to include LTC resident’s perspective by having interviews conducted in dyads with family members and by including observations where verbal communication was not possible. This study also follows the principles of the COREQ, ensuring rigour in the results. The results can be used by physiotherapists working in LTC to guide their decision making around goals to focus on with resident who may have difficulty expressing them. Further, policymakers can use these results to inform development and funding of rehabilitation programs to adequately address the identified goals. A limitation of this study is that we recruited participants from two LTC homes owned by the same organization in Halifax, Nova Scotia. Therefore, the results may represent the values of one organization rather than all LTC homes. Another limitation of this study is the limited number of residents who participated, all of whom were women. Approximately 70% of the Canadian LTC home population is women [32]. Therefore, the findings may not be transferable to men in LTC. Also, the use of dyadic interview format with family members and residents has the limitation of domination, where there is a possibility of acquiescence bias occurring in situations where power dynamics are unevenly distributed within the dyad cohort [27].

In conclusion, we identified that rehabilitation goals for LTC residents living with dementia often focus on quality of life and functional activities and participation in LTC and family activities and events. Thus, rehabilitation in LTC should focus on improving function and quality of life. There is an interrelationship between function and quality of life, where functional goals also contribute to improved quality of life. While some goals focus on improvement in function, maintenance or prevention of decline were also key elements. Future work should ensure rehabilitation interventions are developed relative to individually identified goals, and interventional success is measured in relation to the goal.

### Electronic supplementary material

Below is the link to the electronic supplementary material.

Supplementary Material 1

Supplementary Material 2

## Data Availability

The data analyzed in this study are not publicly available due to privacy and confidentiality restrictions pertaining to person-level health information in Canada. However, the data set creation plan and underlying analytic code are available from the corresponding author on reasonable request.

## Abbreviations

LTC: long–term care
ICF: International Classification of Functioning, Disability, and Health
