# Supplementary material for: Resident-, family-, and staff-identified goals for rehabilitation of long-term care residents with dementia: a qualitative study
Source: BMC Geriatr. 2024 Jan 29;24:108. doi: 10.1186/s12877-024-04674-2 (PMC10825995; doi:10.1186/s12877-024-04674-2)
Supplement: Supplementary file 2 — Supplementary Material 2 [file 12877_2024_4674_MOESM2_ESM.docx]

**Interview guide – Residents**

**CONCEPT: What are your goals for rehabilitation? What parts of rehabilitation are enjoyable/not enjoyable?**

1. **Do you like to move/be active/exercise?**

*Follow-up/probing questions:​*

1. (If says no) Why don’t you like to move/be active/exercise?
2. What makes you happy?
3. (If says yes) What do you like to do when you exercise/move/be active? (ex. walking, dancing, etc.)?
4. How does that make you feel?
5. What can you do to keep doing those things?​

e) What makes it hard for you to do things you like to do?​

**CONCEPT - What are your goals for rehabilitation?​**

1. What do you want to improve/maintain/prevent? (e.g., falls, fractures, ability to walk, transfer, etc)
2. What activities do you like to do? What would like to keep doing?​

**Is there anything else you would like to tell me?**

**Interview Guide – Family Members**

**CONCEPT - What are your family member’s goals for rehabilitation?**

*Follow-up/probing questions:*

1. What would your family member want to improve/maintain/prevent? (e.g., falls, fractures, ability to walk, transfer, etc)
2. What activities does your family member like to do?
3. What would they like to keep doing?

b) What things can we measure to make sure rehabilitation is doing a good job in long-term care?

**CONCEPT - What does quality of life mean for your family member?**

*Follow-up/probing questions:*

a) When you think of quality of life for them, what do you think of? What makes them feel good/happy? How does that make you and them feel?

b) If your family member has good/bad quality of life, what does that look like? What does it mean? What does makes them feel upset/unhappy? What don’t they like? How does that make them feel?

**CONCEPT - What does rehabilitation mean?**

*Follow-up/probing questions:*

1. When you think of rehabilitation for your family member, what do you think of?
2. What makes them happy? What can we do to help keep them doing those things?

b) What types of activities do you think rehabilitation in long-term care should/could do? Do they like to move/be active/exercise? What do they like to do when they exercise/move/be active? How does that make them feel? (If says no) Why don’t they like to move/be active/exercise? How does it make them feel?

c) Why do they think this way about rehabilitation in long-term care?

**CONCEPT - What does function mean to your family member?**

*Follow-up/probing questions:*

a) When they think of function, what do you think of? What keeps them moving/going?

b) If your family member has good/bad function, what does that look like? What does it mean? What makes it hard for them to do things they like to do?

**5. Is there anything else you would like to tell me about rehabilitation in long-term care?**

**Interview Guide – Staff**

**CONCEPT - What are residents’ goals for rehabilitation?**

*Follow-up/probing questions:*

1. What would the resident want to improve/maintain/prevent? (e.g., falls, fractures, ability to walk, transfer, etc)
2. What activities does the resident like to do?
3. What would they like to keep doing?

b) What things can we measure to make sure rehabilitation is doing a good job in long-term care?

**CONCEPT - What does quality of life mean for the resident?**

*Follow-up/probing questions:*

a) When you think of quality of life for them, what do you think of? What makes them feel good/happy? How does that make you and them feel?

b) If the resident has good/bad quality of life, what does that look like? What does it mean? What does makes them feel upset/unhappy? What don’t they like? How does that make them feel?

**CONCEPT - What does rehabilitation mean?**

*Follow-up/probing questions:*

1. When you think of rehabilitation for the resident, what do you think of?
2. What makes them happy? What can we do to help keep them doing those things?

b) What types of activities do you think rehabilitation in long-term care should/could do? Do they like to move/be active/exercise? What do they like to do when they exercise/move/be active? How does that make them feel? (If says no) Why don’t they like to move/be active/exercise? How does it make them feel?

c) Why do they think this way about rehabilitation in long-term care?

**CONCEPT - What does rehabilitation mean?**

*Follow-up/probing questions:*

1. When you think of rehabilitation for the resident, what do you think of?
2. What makes them happy? What can we do to help keep them doing those things?

b) What types of activities do you think rehabilitation in long-term care should/could do? Do they like to move/be active/exercise? What do they like to do when they exercise/move/be active? How does that make them feel? (If says no) Why don’t they like to move/be active/exercise? How does it make them feel?

c) Why do they think this way about rehabilitation in long-term care?

**Semi-structured Field Note Guide**

**1. Describe the physical setting**

*Prompts:*

a) Where were you in the home?

b) Who else was around?

c) What time of day was it? (e.g., morning, afternoon, evening)

d) What other activities were happening?

e) What was the resident doing?

**2. Describe the social setting**

*Prompts:*

a) Who did they interact with?

b) How did they interact?

c) How did they communicate? Describe verbal and non-verbal communication

d) Who did they communicate with?

e) What was the meaning of their interactions?

f) Any comments related to the purpose of the study?

g) What impact did the observer have on the situation?

**3. Ideas, impressions, thoughts about what was observed**

*Prompts:*

a) What was the meaning of their actions/words/expressions?

b) Why do you think you saw what you did?

c) What would you change or think about for future observations?
